# No association between polymorphisms in *PTEN* and primary ovarian insufficiency in a Han Chinese population

**DOI:** 10.1186/s12958-015-0057-5

**Published:** 2015-06-17

**Authors:** Weiwei Zou, Binbin Wang, Jing Wang, Zhiguo Zhang, Xiaofeng Xu, Beili Chen, Xu Ma, Yunxia Cao

**Affiliations:** Reproductive Medicine Center, Department of Obstetrics and Gynecology, The First Affiliated Hospital of Anhui Medical University, Hefei, China; Institute of Reproductive Genetics, Anhui Medical University, Hefei, China; Anhui Provincial Engineering Technology Research Center for Biopreservation and Artificial Organs, Hefei, China; Center for Genetics, National Research Institute for Family Planning, 12, Dahuisi Road, Haidian Beijing, 100081 China; Peking Union Medical College, Beijing, China; World Health Organization Collaborating Centre for Research in Human Reproduction, Beijing, China

**Keywords:** Primary ovarian insufficiency, PTEN, Polymorphisms, Chinese Han population

## Abstract

**Background:**

The aim of our study was to investigate the possible relationship between polymorphisms in PTEN (the phosphatase and tensin homolog located on chromosome ten in humans) and POI (primary ovarian insufficiency) in Chinese women.

**Methods:**

Seven tag SNPs (single nucleotide polymorphisms) - rs1234219, rs1903858, rs2299939, rs35352882, rs17107001, rs2299941 and rs12572106 - were chosen from the CHB (Han Chinese people in Beijing, China) HapMap database. MALDI-TOF-MS (matrix-assisted laser desorption/ionisation time-of-flight mass spectrometry) was used to detect the genotype distribution of the seven SNPs among 148 POI patients and 230 controls.

**Results:**

No statistically significant difference was found in an association analysis of the seven SNPs in the allele frequencies, genotype frequencies, or haplotype distributions.

**Conclusions:**

In summary, this study explored the relationship between polymorphisms in PTEN and POI in a Han Chinese population and suggests that polymorphisms in PTEN may not be associated with a risk of POI.

## Background

POI (primary ovarian insufficiency) is characterised by reduced ovarian function in women under 40 years of age. It results from a decrease in the number of ovarian follicles, follicle exhaustion or follicle insensitivity to gonadotropins [1]. It has been reported that approximately 1 % of women under 40 years of age with a normal karyotype are affected by this disease [2]. In the vast majority of POI patients, its aetiology is unknown [3], but it is thought to have a genetic basis [2].

In mammals, the initial number of primordial follicles in the ovary is fixed early in life. Before puberty begins, partial primordial follicles in dormancy are activated and develop to the antral follicle stage. At the onset of puberty, antral follicles either develop into mature follicles or become apoptotic. The remaining primordial follicles remain in a dormant state and wait for further development or undergo apoptosis directly from dormancy [4]. The gradual and natural reduction and ultimate depletion in the number of primordial follicles leads to a gradual deterioration of the ovary, after which the female enters menopause [4]. How can primordial follicles maintain such a long dormant state during a female’s entire reproductive life? Previous studies have found that some signal molecules, such as PTEN (the protein encoded by the phosphatase and tensin homolog located on chromosome ten in humans), are relevant to this process [5]. Deletion of *Pten* in mouse oocytes results in the premature activation and eventually exhaustion of the global primordial follicles in early adulthood and thus leads to premature ovarian insufficiency [5].

PTEN is a tumour suppressor that has dual-phosphatase activity. Down-regulation of its expression or dysfunction of the *PTEN* (the phosphatase and tensin homolog located on chromosome ten in humans) is closely related to the occurrence of numerous human tumours [6]. PTEN can influence cell apoptosis, invasion and angiogenesis through its down-regulation of the PI3K (phosphoinositide-3 kinase) signal pathway, and it can therefore inhibit the survival and invasion of tumour cells [6]. PTEN also plays an important role in stabilising chromosomes and participates in controlling DSB (DNA double-strand break) repair [7].

Mutations of *Pten* in ES (embryonic stem) cells have a negative influence on early embryonic differentiation in mice and lead to early embryonic death [8]. This suggests that *Pten* is essential for the development of mouse embryos [8]. *Pten*-knockout mice consistently exhibit higher levels of FSH and LH, reduced ovarian function and female infertility [5]. Scientists have also cultured ovaries from newborn mice in vitro in the presence of a PTEN inhibitor and PI3K activator and found that primordial follicles were activated from dormancy [9]. They subsequently cultured human ovarian cortical fragments with the PTEN inhibitor; primordial follicles were activated and demonstrated the ability to develop into mature follicles [9].

Given the above findings, we suspected that functional deficiency or dysfunction of *PTEN* in humans might result in damaged ovarian functions and lead to primary ovarian insufficiency. We therefore explored a possible relationship between polymorphisms of *PTEN* and POI in Chinese women.

## Methods

This study was approved by the institutional ethical committee of Anhui Medical University. All participants were informed of and consented to the experiment.

### Subjects

In total, 378 Chinese women, including 148 POI patients (mean age ± SD, 29.7 ± 6.1 years) (E2 96.1 ± 82.1 pmol/L, FSH 74.1 ± 30.6 mIU/mL, LH 37.6 ± 19.3 mIU/mL) and 230 controls (mean age ± SD, 30.2 ± 3.7 years) (E2 187.3 ± 92.5 pmol/L, FSH 6.5 ± 1.6 mIU/mL, LH 5.3 ± 3.1 mIU/mL), were recruited from the First Affiliated Hospital, Anhui Medical University, China. We chose POI patients who had had amenorrhea for more than 6 months and had normal diploid karyotypes and serum FSH levels > 30 IU/L [10]. We excluded patients who had undergone pelvic surgery, had been diagnosed with an autoimmune disease, or had ever received chemotherapy or radiotherapy. All of the controls were healthy Chinese women with natural menstrual cycles, normal FSH values and no evidence of diseases affecting normal ovarian function (including large ovarian or ovarian endometriosis cysts, severe tubal lesions, hypothalamus or pituitary tumours, obesity, abnormal adrenal function, thyroid disease, etc.). The approval and consent of the institutional ethics committee of Anhui Medical University was obtained for this experiment.

Considering the pathogenic mechanism of *PTEN*, the POI patients were divided into several groups: those with primary amenorrhea and secondary amenorrhea and early amenorrhea (including 22 primary amenorrhea patients and 4 patients with amenorrhea that only experienced menarche, but developed amenorrhea immediately after the initial menses) and late amenorrhea (secondary amenorrhea, excluding 4 patients with amenorrhea that only experienced menarche, but developed amenorrhea immediately after the initial menses).

### Methods and genetic analysis

A QIAamp DNA Blood Mini Kit (Qiagen, Germany) was used for genomic DNA purification from peripheral blood. Seven tag SNPs - rs1234219, rs1903858, rs2299939, rs35352882, rs17107001, rs2299941 and rs12572106 - were chosen from the CHB (Han Chinese people in Beijing, China) HapMap database [11]. The criteria for selection were tag SNPs with a MAF (minor allele frequency) > 0.05 and r^2^ > 0.8. MALDI-TOF-MS (matrix-assisted laser desorption/ionisation time-of-flight mass spectrometry) (Illumina, US) was used to detect the genotypes of the seven SNPs in the extracted samples from the POI patients and controls. To control the quality, we performed the detections repeatedly in both standard samples and experimental samples.

### Statistical analysis

Comparative analyses of the allele frequencies and genotype frequencies of rs1234219, rs1903858, rs2299939, rs35352882, rs17107001, rs2299941 and rs12572106 between POI patients and controls were performed with Pearson’s chi-squared tests using SPSS 13.0 software. We used Fisher’s exact test if the expected count was less than 5 in any of the cells. Odds ratios (ORs) and their matching 95 % confidence intervals (CIs) were computed using logistic regression analysis via SPSS 13.0 software. The HWE (Hardy-Weinberg equilibrium) of each SNP polymorphism in POI patients and controls was evaluated using Pearson’s chi-squared tests, and we also applied Fisher’s exact test if the expected count was less than 5 in any of the cells. LD (Linkage disequilibrium), haplotype association analysis and permutation tests were performed using HaploView 4.2 software. Haplotypes with frequencies less than 0.05 were excluded. To avoid statistical errors, we performed multiple comparisons using Bonferroni correction. Data with p-values < 0.05 were considered statistically significant.

## Results

**Fig. 1:**
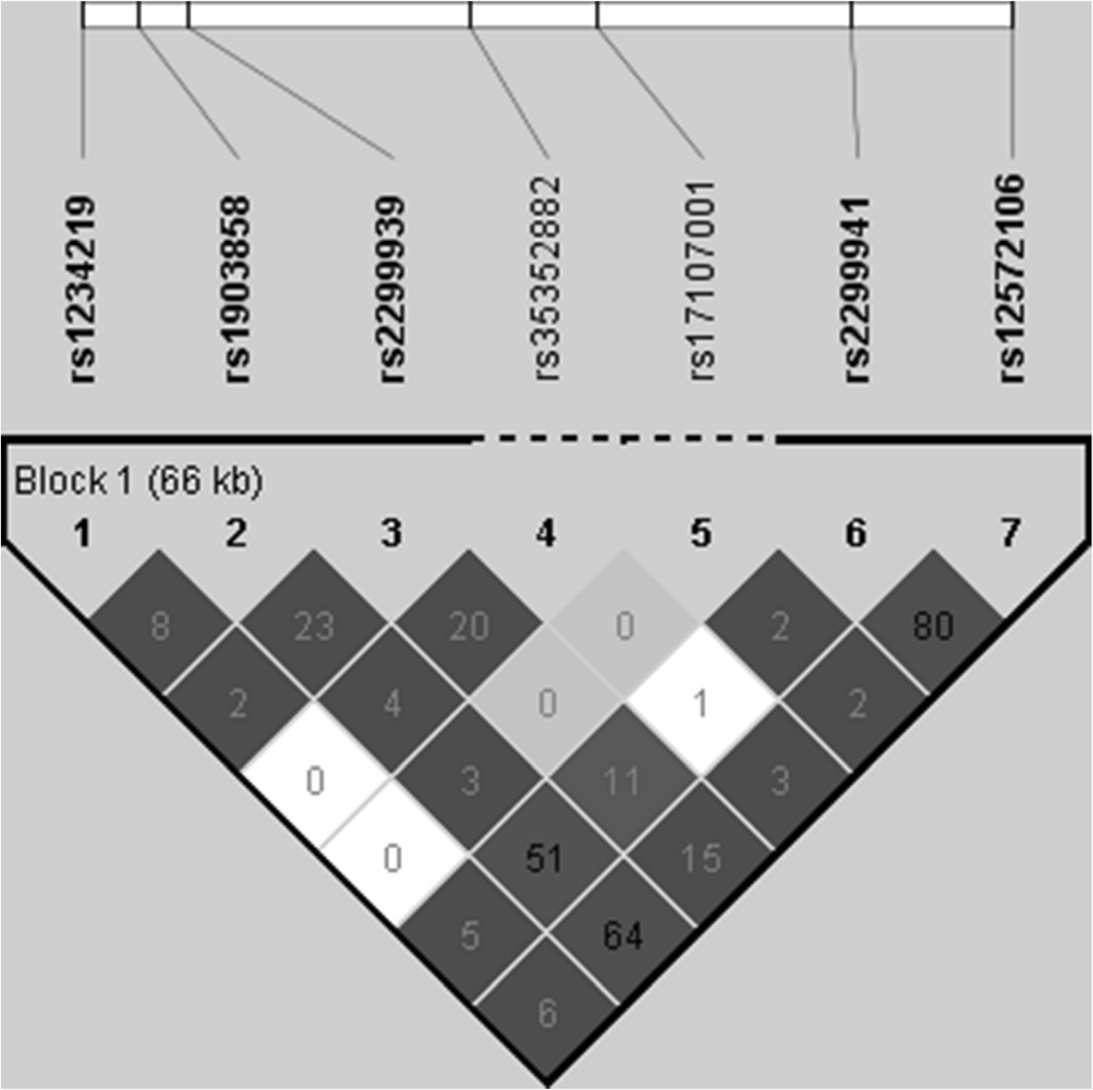
Linkage disequilibrium LD plot of the seven SNPs (rs1234219, rs1903858, rs2299939, rs35352882, rs17107001, rs2299941 and rs12572106) in the *PTEN* region are shown. Calculation of the r^2^ values, shown in each square, was based on data from 230 controls using HaploView 4.2 software. While two of the SNPs (rs12572106 and rs2299941) show a high LD, the other SNPs show a low LD (r^2^ < 0.8). rs35352882 and rs17107001 were excluded from the haplotype analyses and are therefore connected by a dotted line

The LD plot of the seven SNPs (single nucleotide polymorphisms) in the *PTEN* region is shown in Fig. 1. Only two SNPs (rs12572106 and rs2299941) were in strong LD. The seven SNPs are located in the introns of *PTEN*.

The allele frequencies and association analysis of the seven SNPs are shown in Table 1. In both POI patients and controls, the seven SNPs of PTEN were all in an HWE state. The allele frequencies of rs2299939 showed a statistically significant difference (*p* <0.05) between POI patients and controls. However, after Bonferroni correction, no significant difference was found for any of the SNPs.

**Table 1:** The allele frequencies of the seven SNPs in *PTEN* between POI patients and controls

SNP	Alleles	Associated allele	HWE cases	HWE controls	MAF cases (*n* = 148)	MAF controls (*n* = 230)	OR	95 % CI	Value	*p*	Bonferroni correction
rs1234219	T/C	C	0.95	0.72	0.084	0.084	1.010	0.596–1.711	0.001	0.971	1.000
rs1903858	C/T	C	0.60	0.90	0.446	0.509	0.777	0.579–1.043	2.834	0.092	0.644
rs2299939	C/A	C	0.45	0.16	0.146	0.206	0.661	0.445–0.982	4.230	0.040	0.280
rs35352882	T/C	C	0.58	0.51	0.044	0.042	1.047	0.509–2.153	0.015	0.901	1.000
rs17107001	G/T	T	0.64	0.63	0.037	0.031	1.208	0.541–2.697	0.212	0.645	1.000
rs2299941	A/G	G	0.68	0.62	0.378	0.354	1.111	0.820–1.505	0.460	0.498	1.000
rs12572106	T/C	C	0.74	0.44	0.443	0.389	1.245	0.925–1.676	2.091	0.148	1.000

The allele frequencies were performed with Pearson’s chi-squared test or Fisher’s exact test using SPSS 13.0 software. The allele frequencies of rs2299939 showed no significant difference after Bonferroni correction between POI patients and the controls

The genotype frequencies of the seven SNPs are shown in Table 2. There was no statistically significant difference for any of the SNPs between POI patients and controls in genotype frequencies.

**Table 2:** The genotype frequencies of the seven SNPs in *PTEN* between POI patients and controls

SNP	Genotypes, n				Value	p	OR (95 % CI)
rs1234219	T/T	T/C	C/C	Missing			
Cases	124	23	1	0	0.201	0.950	0.770 (0.069–8.584)
Controls	191	34	2	3			1.042 (0.586–1.852)
rs1903858	C/C	C/T	T/T	Missing			
Cases	47	70	31	0	2.889	0.236	1.367 (0.837–2.233)
Controls	55	112	59	4			0.841 (0.496–1.425)
rs2299939	C/C	C/A	A/A	Missing			
Cases	106	39	2	1	5.319	0.070	4.719 (1.043–21.353)
Controls	146	67	13	4			3.784 (0.811–17.651)
rs35352882	T/T	T/C	C/C	Missing			
Cases	135	13	0	0	0.016	0.899	1.049 (0.502–2.195)
Controls	207	19	0	4			
rs17107001	G/G	G/T	T/T	Missing			
Cases	137	11	0	0	0.220	0.639	0.822 (0.363–1.864)
Controls	212	14	0	4			
rs2299941	A/A	A/G	G/G	Missing			
Cases	56	72	20	0	0.854	0.652	1.080 (0.568–2.052)
Controls	96	100	30	4			0.875 (0.455–1.684)
rs12572106	T/T/	T/C	C/C	Missing			
Cases	47	71	30	0	2.048	0.359	1.501 (0.825–2.730)
Controls	87	102	37	4			1.288 (0.808–2.054)

The genotype frequencies were performed with Pearson’s chi-squared test or Fisher’s exact test using SPSS 13.0 software. There was no significant difference between POI patients and the controls

The haplotype distributions of five SNPs are shown in Table 3. Only two genotypes were found for two SNPs (rs35352882 and rs17107001), and the MAF was under 0.05. Because the inclusion of those two SNPs could result in a considerable statistical error, we analysed only the other five SNPs for haplotype distributions. The p-value of T-T-A-A-T showed a statistically significant difference (*p* < 0.05), but there was no significant difference after 10^4^ permutation tests.

**Table 3:** The haplotype distributions of *PTEN* polymorphisms in POI patients and controls

Haplotype	Total frequency	Cases frequency	Controls frequency	*p*-value	*p*-value from permutation
T-C-C-G-C	0.358	0.378	0.345	0.355	0.820
T-T-C-A-T	0.302	0.301	0.302	0.959	1.000
T-T-A-A-T	0.181	0.145	0.204	0.042	0.175
C-C-C-A-T	0.084	0.084	0.084	0.971	1.000

The haplotype association analysis was performed using the HaploView 4.2 software. The p-value of T-T-A-A-T showed a statistically significant difference, but there was no significant difference after 10^4^ permutation tests

There was no statistically significant difference in the allelic and genotypic distributions between primary and secondary amenorrhea, early and late amenorrhea and the control groups after Bonferroni correction (Table 4, Table 5).

**Table 4:** The association analysis of the seven SNPs among the primary amenorrhea, secondary amenorrhea and control groups

SNP	Genotypes, n				Value	*p*	Alleles		Value	*P*	Bonferroni correction
rs1234219	T/T	T/C	C/C	Missing			T	C			
Primary amenorrhea	19	3	0	0	0.659	0.989	41	3	0.093	0.976	1.000
Secondary amenorrhea	105	20	1	0			230	22			
Controls	191	34	2	3			416	38			
rs1903858	C/C	C/T	T/T	Missing			C	T			
Primary amenorrhea	3	14	5	0	7.452	0.114	20	24	4.883	0.087	0.609
Secondary amenorrhea	44	56	26	0			144	108			
Controls	55	112	59	4			222	230			
rs2299939	C/C	C/A	A/A	Missing			C	A			
Primary amenorrhea	19	3	0	0	6.694	0.132	41	3	6.346	0.042	0.294
Secondary amenorrhea	87	36	2	1			210	40			
Controls	146	67	13	4			359	93			
rs35352882	T/T	T/C	C/C	Missing			T	C			
Primary amenorrhea	20	2	0	0	0.179	1.000	42	2	0.180	0.956	1.000
Secondary amenorrhea	115	11	0	0			241	11			
Controls	207	19	0	4			433	19			
rs17107001	G/G	G/T	T/T	Missing			G	T			
Primary amenorrhea	21	1	0	0	0.478	0.832	43	1	0.451	0.835	1.000
Secondary amenorrhea	116	10	0	0			242	10			
Controls	212	14	0	4			438	14			
rs2299941	A/A	A/G	G/G	Missing			A	G			
Primary amenorrhea	9	11	2	0	1.205	0.886	29	15	0.774	0.679	1.000
Secondary amenorrhea	47	61	18	0			155	97			
Controls	96	100	30	4			292	160			
rs12572106	T/T	T/C	C/C	Missing			T	C			
Primary amenorrhea	7	13	2	0	4.244	0.370	27	17	2.766	0.251	1.000
Secondary amenorrhea	40	58	28	0			138	114			
Controls	87	102	37	4			276	176			

The allele and genotype frequencies among the primary amenorrhea, secondary amenorrhea and control groups were performed with Pearson’s chi-squared test or Fisher’s exact test using SPSS 13.0 software. There was no statistically significant difference in the allelic and genotypic distributions between the primary amenorrhea, secondary amenorrhea and control groups after Bonferroni correction

**Table 5:** The association analysis of the seven SNPs among the early amenorrhea, late amenorrhea and control groups

SNP	Genotype, n (%)				Value	*p*	Alleles		Value	*P*	Bonferroni correction
rs1234219	T/T	T/C	C/C	Missing		0.945	T	C		0.810	1.000
Early amenorrhea	23	3	0	0	0.820		49	3	0.418		
Late amenorrhea	101	20	1	0			222	22			
Controls	191	34	2	3			416	38			
rs1903858	C/C	C/T	T/T	Missing		0.195	C	T		0.168	1.000
Early amenorrhea	5	16	5	0	6.057		26	26	3.572		
Late amenorrhea	42	54	26	0			138	106			
Controls	55	112	59	4			222	230			
rs2299939	C/C	C/A	A/A	Missing		0.143	C	A		0.044	0.308
Early amenorrhea	22	4	0	0	6.515		48	4	6.267		
Late amenorrhea	84	35	2	1			203	39			
Controls	146	67	13	4			359	93			
rs35352882	T/T	T/C	C/C	Missing		0.955	T	C		0.957	1.000
Early amenorrhea	24	2	0	0	0.101		50	2	0.095		
Late amenorrhea	111	11	0	0			233	11			
Controls	207	19	0	4			433	19			
rs17107001	G/G	G/T	T/T	Missing		0.704	G	T		0.711	1.000
Early amenorrhea	25	1	0	0	0.679		51	1	0.840		
Late amenorrhea	112	10	0	0			234	10			
Controls	212	14	0	4			438	14			
rs2299941	A/A	A/G	G/G	Missing		0.731	A	G		0.776	1.000
Early amenorrhea	9	15	2	0	2.056		33	19	0.506		
Late amenorrhea	47	57	18	0			151	93			
Controls	96	100	30	4			292	160			
rs12572106	T/T	T/C	C/C	Missing		0.527	T	C		0.352	1.000
Early amenorrhea	7	15	4	0	3.189		29	23	2.091		
Late amenorrhea	40	56	26	0			136	108			
Controls	87	102	37	4			276	176			

The allele and genotype frequencies among the early amenorrhea, late amenorrhea and control groups were performed with Pearson’s chi-squared test or Fisher’s exact test using SPSS 13.0 software. There was no statistically significant difference in the allelic and genotypic distributions between the early amenorrhea, late amenorrhea and control groups after Bonferroni correction

## Discussion

There are two mechanisms that are involved in the occurrence of POI: follicle dysfunction and follicle premature exhaustion [3]. Follicle premature exhaustion indicates the accelerated apoptosis of primordial follicles or premature activation of primordial follicles. Therefore, the survival of primordial follicles is necessary for normal ovarian function. Early researchers inferred that there are inhibitory factors in intact ovarian tissue that maintain the majority of primordial follicles in a dormant state [12]. Previous studies have demonstrated that the PI3K signal pathway is one of the crucial molecular mechanisms that are involved in the survival, dormancy and apoptosis of mammalian primordial follicles [13]. Multiple signal molecules of this pathway were recognised as either stimulating factors (such as Ribosomal protein S6) or inhibitory factors (such as tuberous sclerosis complex 1 and tuberous sclerosis complex 2) in maintaining the balance of the activation and dormancy of mammalian primordial follicles [13, 14]. PTEN down-regulates the PI3K signal pathway [13]. The functional deficiency or dysfunction of *Pten* leads to continuous and excessive activation of the downstream signal pathway and results in excessive activation and depletion of primordial follicles, ultimately leading to ovarian failure in mice [13, 14]. Before the complete depletion of primordial follicles, *Pten* mutant mice undergo normal ovulation and generate normal offspring [15, 16].

In the present study, we sought to study the relationship between seven SNPs (rs1234219, rs1903858, rs2299939, rs35352882, rs17107001, rs2299941 and rs12572106) in *PTEN* and POI in 148 POI patients and 230 controls. We performed an association analysis of the seven SNPs with regard to their allele frequencies, genotype frequencies and haplotype distributions. Rs2299939 showed a statistically significant difference in allele frequency, but the difference disappeared after Bonferroni correction. Haplotype T-T-A-A-T showed an association with POI, but it too disappeared after 10^4^ permutation tests.

POI is a complex disease that involves multiple genes. Although many genes have been confirmed as being involved in the development of POI [2], the cause of most cases remains unidentified. Therefore, many investigators have devoted their research to identifying the aetiology of POI. Because previous studies have found that the deletion of *PTEN* in oocytes leads to the failure of ovarian function in mice [5], we sought to determine whether *PTEN* is involved in the incidence and development of human POI. We did not find an association between POI and any risk alleles, genotypes or haplotypes. There are several possible explanations for these results. First, *PTEN* has never been demonstrated to be associated with human POI. In addition, specific differences between mice and humans may account for genetic differences in the *PTEN* region. Although researchers have found that compared to mice more primordial follicles were activated in human ovarian cortical fragments after treatment with a *PTEN* inhibitor [9], they did not analyse the statistical significance of the difference. In addition, that study’s sample size may not have been sufficiently large. In the previous study, the increased gonadotropin concentrations, morphological changes of the ovary and the depletion of primordial follicles began to appear 12 weeks postnatal in PTEN mutant mice. In humans, the period from birth to puberty may last over 10 years. If the gene mutation or abnormal function of PTEN occurs in the embryonic or early life, it is likely that ovarian primordial follicles may be activated and exhausted before puberty, which may present as primary amenorrhea. If the gene mutation or abnormal function of PTEN occurs after puberty, it behaves as secondary amenorrhea. Therefore, we divided the cases into primary and secondary amenorrhea and early and late amenorrhea and compared the patients with the controls. If we obtained meaningful results, we could affirm the association between PTEN and POI and could also infer the approximate time of the PTEN mutation. Rs2299939 showed a statistically significant difference between these groups in allele frequency, but the difference disappeared after Bonferroni correction. The primary amenorrhea sample size was too small. A larger sample size of primary amenorrhea POI patients may yield statistically significant results, especially considering the promising rs2299939 allele and T-T-A-A-T haplotype.

Researchers analysed the PCR products of the *PTEN* encoding region via direct sequencing in 20 idiopathic POI patients and 20 controls, but found no meaningful mutation [17]. We hypothesise that a larger sample size may improve results, and in population genetics, many different results are due to the varied composition of the studied populations. Chinese scientists had already performed a mutation analysis of nine exons of *PTEN* in 161 Chinese women with POI but did not observe any mutations or variants [18]. The sample size of their study was similar to the sample size used in the present study, and they researched *PTEN* from various angles, performing mutational analysis of exons and conducting an association study of intronic SNPs. The two studies mutually remedy their respective defects and together indicate that PTEN may not participate in the pathogenesis of POI. In addition, the POI patients in the two studies were all secondary amenorrhea patients. Thus, we need to confirm the results in a larger sample of patients with primary amenorrhea.

## Conclusions

In summary, this study investigated the relationship between polymorphisms of *PTEN* and POI in a Chinese Han population. Our findings suggest that *PTEN* may not be a common pathogenic gene for POI in humans. However, further studies in a larger sample size or functional studies of *PTEN* are needed to verify these results.

## Abbreviations

CHB: (Han Chinese people in Beijing, China)
CI: (confidence interval)
DSB: (DNA double-strand break)
ES: (Embryonic stem)
HWE: (Hardy-Weinberg equilibrium)
LD: (Linkage disequilibrium)
MALDI-TOF-MS: (Matrix-assisted laser desorption/ionisation time-of-flight mass spectrometry)
OR: (Odds ratio)
PI3K: (Phosphoinositide-3 kinase)
POI: (primary ovarian insufficiency)
*PTEN*: (the phosphatase and tensin homolog located on chromosome ten in humans)
PTEN: (The protein encoded by the phosphatase and tensin homolog located on chromosome ten in humans)
SNP: (Single nucleotide polymorphism)
